# Effects of immersive virtual reality on sensory overload in a random sample of critically ill patients

**DOI:** 10.3389/fmed.2023.1268659

**Published:** 2023-10-04

**Authors:** Aileen C. Naef, Stephan M. Gerber, Michael Single, René M. Müri, Matthias Haenggi, Stephan M. Jakob, Marie-Madlen Jeitziner, Tobias Nef

**Affiliations:** ^1^Gerontechnology and Rehabilitation Group, ARTORG Center For Biomedical Engineering Research, University of Bern, Bern, Switzerland; ^2^Department of Neurology, Inselspital, Bern University Hospital, University of Bern, Bern, Switzerland; ^3^Department of Intensive Care Medicine, Inselspital, Bern University Hospital, University of Bern, Bern, Switzerland; ^4^Department of Public Health (DPH), Faculty of Medicine, Institute of Nursing Science (INS), University of Basel, Basel, Switzerland

**Keywords:** virtual reality, intensive care unit, critical care, relaxation, feasibility

## Abstract

**Background:**

Sensory overload and sensory deprivation have both been associated with negative health outcomes in critically ill patients. While there is a lack of any clear treatment or prevention strategies, immersive virtual reality is a promising tool for addressing such problems, but which has not been repetitively tested in random samples. Therefore, this study aimed to determine how critically ill patients react to repeated sessions of immersive virtual reality.

**Methods:**

This exploratory study was conducted in the mixed medical–surgical intermediate care unit of the University Hospital of Bern (Inselspital). Participants (*N* = 45; 20 women, 25 men; age = 57.73 ± 15.92 years) received two immersive virtual reality sessions via a head-mounted display and noise-canceling headphones within 24 h during their stay in the unit. Each session lasted 30-min and showed a 360-degree nature landscape. Physiological data were collected as part of the participants’ standard care, while environmental awareness, cybersickness, and general acceptance were assessed using a questionnaire designed by our team (1 = not at all, 10 = extremely).

**Results:**

During both virtual reality sessions, there was a significant negative linear relationship found between the heart rate and stimulation duration [first session: *r*(43) = −0.78, *p* < 0.001; second session: *r*(38) = −0.81, *p* < 0.001] and between the blood pressure and stimulation duration [first session: *r*(39) = −0.78, *p* < 0.001; second session: *r*(30) = −0.78, *p* < 0.001]. The participants had a high comfort score [median (interquartile range {IQR}) = 8 (7, 10); mean = 8.06 ± 2.31], did not report being unwell [median (IQR) = 1 (1, 1); mean = 1.11 ± 0.62], and were not aware of their real-world surroundings [median (IQR) = 1 (1, 5); mean = 2.99 ± 3.22].

**Conclusion:**

The subjectively reported decrease in environmental awareness as well as the decrease in the heart rate and blood pressure over time highlights the ability of immersive virtual reality to help critically ill patients overcome sensory overload and sensory deprivation. Immersive virtual reality can successfully and repetitively be provided to a randomly selected sample of critically ill patients over a prolonged duration.

## 1. Introduction

In the intensive care unit (ICU), patients can experience a spectrum of sensory inputs that are known to negatively affect patient outcomes (1–3). These inputs range from a near-constant exposure to lights, sounds, people, and smells to a noticeable lack of changing sensory inputs, which are known as sensory overload and sensory deprivation, respectively (3–8). To date, there is no clear treatment or prevention strategy to protect patients from these extremes, warranting further research to help create a more healing environment (6–8).

To achieve this, specific aspects in the ICU that make it a stressful setting must be addressed (1, 4, 9). Specifically, the continual, loud, and meaningless noise along with the near-constant presence of light are thought to be among the main contributors of stress and is often referred to as sensory overload (2–4, 9). These inputs also play a role in the disruption of the circadian rhythm (10). This disruption has been found to be a factor in many negative health outcomes associated with an ICU stay, such as confusion, impaired memory, and increased mortality (2, 4, 10). On the opposite end of the spectrum, sensory deprivation is considered equally harmful and is associated with a lack of external stimuli (3, 11). It can affect patients who are in isolation wards within the ICU and are allowed only minimal in-person contact with other individuals (3, 4, 12). This scenario can contribute to the development of cognitive dysfunction, including hallucinations, irritability, and difficulty in concentrating (3, 13).

Despite the known negative consequences of sensory overload and sensory deprivation, current prevention and treatment methods are limited. Sensory overload is currently prevented by decreasing or creating more targeted exposure to environmental factors, such as light and noise, while limiting interactions not related to patient care (3, 14). This goal is achieved by using techniques such as the use of seclusion rooms or eye masks and ear plugs to promote sleep (3, 15, 16). Alternately, methods for targeting sensory deprivation in the ICU include scheduling times for staff to be present, encouraging family members to communicate via a telephone, and stimulating the senses (e.g., music therapy, aromatherapy) (3). Therefore, combining a technique to block external sensory stimuli while providing a calmer and controlled sensory input virtually could be a valuable technique for addressing sensory overload and sensory deprivation within the ICU. This is supported by work in healthy individuals that found the greatest relaxation effect was induced when participants were provided with combined audiovisual inputs, thereby effectively blocking the real-world sensory inputs (17).

To our knowledge, limited studies have investigated the effect and acceptability of repetitive virtual reality (VR) sessions in a randomly selected sample of critically ill patients. Past studies have used a variety of methods to determine the usability of such technology, the acceptance among staff and patients, the required setup, and any negative outcomes (17–20). Specifically, Gerber et al. showed the feasibility of using immersive VR in the ICU as well as providing initial work in patients; however, the VR exposure was short, and all participants were patients pre-scheduled to undergo heart surgery and thus shown the VR videos prior to their ICU stay (18). Furthering their work, Jawed et al. showed longer immersive VR videos to ICU patients who were not previously exposed to VR (20). However, the selection criteria for their participants were not clear, but the setup used suggested that the authors used selective sampling (20). The chosen sample of patients is an important factor to consider since sensory overload and sensory deprivation can affect any patient in the ICU. Therefore, it is essential to have a technique that can be applied to a representative ICU sample without selective recruitment. Jawed et al. also did not provide any information about the physiological effects of the VR exposure, which is an important aspect owing to the connection between sensory overload or sensory deprivation and the stressful ICU environment (20). It is also relevant to examine the physiological effects of VR due to the critical health status of patients and to ensure that no potentially harmful outcomes are evoked. Previous work has also failed to clearly investigate the feasibility of providing repeated sessions of immersive VR. Such investigation is important to evaluate the reproducibility of results while ensuring that there is limited bias introduced by the novelty of the setup. Moreover, repeated use of VR systems can provide relevant information about the convenience and efficacy of deploying such systems in the busy ICU setting.

Accordingly, the current study aimed to investigate the use of immersive VR in the ICU as a possible strategy to achieve a healing environment while protecting patients, and to determine how critically ill patients react to repeated immersive VR sessions. Previous work examining immersive VR exposure in healthy individuals and critically ill patients led us to hypothesize that VR would evoke a physiological change among patients and would be able to be repeatedly deployed and well accepted, while subjectively decreasing users’ awareness of their real-world surroundings.

## 2. Methods

### 2.1. Study setting and participants

This exploratory, non-randomized study was conducted from January 17, 2022, to May 13, 2022, in the department of intensive care medicine at the University Hospital of Bern (Inselspital). The department is made up of both an ICU and an intermediate care unit (IMCU), treating both medical and surgical patients. Depending on treatment requirements and care needs, patients are transferred fluidly between the two units. All patients in the IMCU were screened for participation in the study. A random sample of eligible patients was then approached by a study team member, who explained the study protocol verbally. A written information sheet was provided to all eligible patients, who were then given time to consider participation in the study. Upon agreement to participate, written informed consent from each patient was obtained. This study was approved by the Ethics Committee of the Canton of Bern, Switzerland (KEK-2020-00039b) and was registered on ClinicalTrials.gov (NCT05380700, registered on May 19th, 2022, retrospectively registered). All study aspects were carried out following the current version of the Declaration of Helsinki.

The inclusion criteria were a minimum age of 18 years, ability to speak German or French, absence of self-reported severe visual or auditory impairments (i.e., normal or corrected to normal), and estimated length of stay of more than 24 h. The exclusion criteria were epilepsy, COVID-19, neurosurgical patient status with removed skullcap, external ventricular drainage, and other intracranial pressure probes. All patients hospitalized in the IMCU were screened daily from Monday to Friday for study eligibility. As the effect size of this intervention in this patient population was unknown, no power calculation was conducted to determine the sample size. The sample size was, therefore, based on expanding what was previously been done in the literature (18, 20). For these reasons, a sample size of 40 participants completing both VR sessions was selected. Participant recruitment and enrollment continued until this sample size was achieved.

### 2.2. Study design and procedure

Once the participants were enrolled in the study, the study was either started immediately or delayed to try to ensure that the 30-min recording could occur uninterrupted. This strategy accommodated scheduled activities, such as medical interventions, scans, physiotherapies, meals, or family visits.

Initially, the participant demographics were collected using a questionnaire and from the patient data management system. Subsequently, the first of two VR sessions was commenced, with the two sessions completed within 24 h of study enrollment. Only one session was initially performed to assess the feasibility of repeated VR stimulation in the ICU and patients’ willingness to participate in a second session. The VR sessions lasted up to 30 min in line with previous work that has shown a relaxation effect in sessions lasting 30 min (17). The intended goal of 30 min was always communicated to the participants. However, we considered 20 min as a successful-stimulation duration owing to logistic reasons, such as the busy nature of the unit, time-sensitive patient care interventions, and short length of stay, which can make longer VR sessions difficult. Any recordings that did not last a minimum of 20 min were considered incomplete and were not included in the final analysis. The participants who did not meet the minimum recording time during the first session were considered to have an unsuccessful session and did not receive a second VR session.

Following each successful VR session, the participants were asked to complete a general questionnaire about their experience wearing the VR device (e.g., comfort, feeling unwell, falling asleep) and awareness of the real world (e.g., noise, light, people) during the intervention (Supplementary Table S1, Additional File 1) (17). The questions were developed by the study team and answered using a 10-point Likert scale; questions were based on previously validated questionnaires (1 = not at all, 10 = extremely) (21, 22). Pain scores were collected immediately prior to, immediately after, and 30 min after each VR session using the validated Numeric Rating Scale (23).

The participants were also asked immediately and 30 min after the sessions whether they felt unwell. The patient database was used to verify that no adverse or serious adverse events (e.g., nausea, significant change in medical state) were reported within 2 h following the VR sessions. Information regarding the medications provided to the participants within 5 h prior to each VR session until the end of each session was collected and grouped (i.e., medications affecting the blood pressure, heart rate, or pain score).

During hospitalization, the participants’ physiological functions were recorded using a monitoring system (Carescape Monitor B650, GE Healthcare, Little Chalfont, Buckinghamshire, UK). The heart rate was recorded via three-lead electrocardiogram. The blood pressure was measured either invasively with an intra-arterial catheter or non-invasively with an arm cuff as part of the patients’ standard care. While the invasive method allowed continuous measurements, the non-invasive method recorded values every 2–5 min.

### 2.3. Stimulation material and apparatus

**Figure 1:**
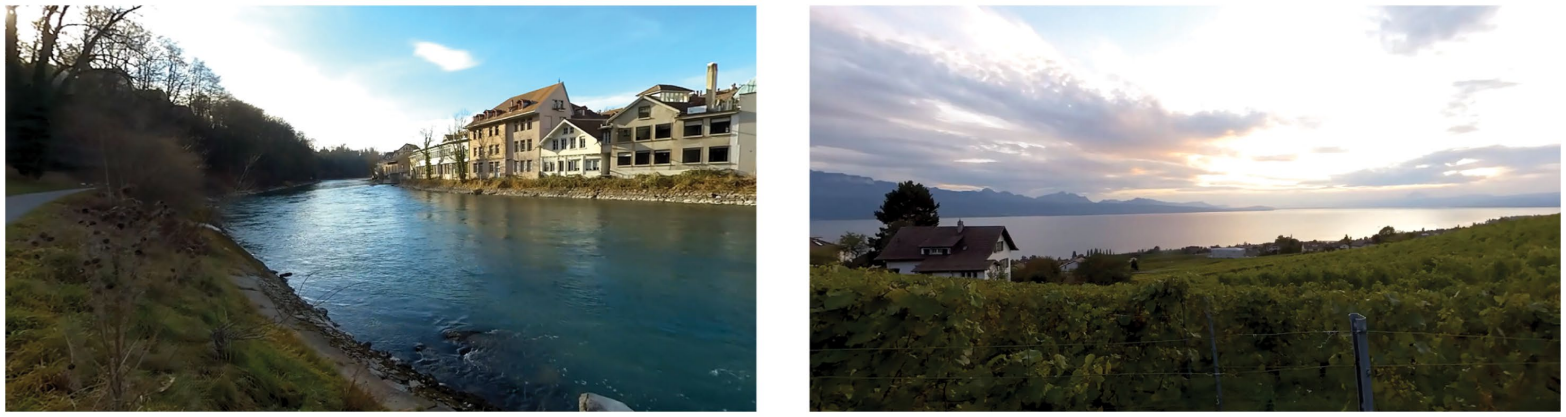
Scenes shown during the virtual reality sessions.

Two 360° videos with a duration of 30 min were used, with the participants seeing each video once. The order in which the videos were shown to the participants was randomized. The content was recorded using a 360° camera (Insta360 Pro 2, Insta360, Shenzhen, Guangdong, China). The videos depicted two locations within Switzerland, both of which aimed to show a calm nature scene and were non-looping. One video showed a view of a river from a riverbank, and the other showed a view from a vineyard overlooking a lake (Figure 1). Commercially available (Listening Earth, Newstead, Queensland, Australia) nature sounds (e.g., water flowing, birds chirping) were used to overlay the videos, with different sounds used per video. Nature videos with corresponding sounds were chosen as the desired content based on a study conducted with individuals previously hospitalized in the ICU and nursing experts (14). Moreover, the choice is in line with the literature showing that nature can induce a relaxation response even when shown virtually (24–29).

The visual content was presented using a head-mounted display (Pico G2 4K; Pico Interactive, San Francisco, California, USA). The device was untethered—there was no need for cables or external devices to run the content. The videos were played directly from the device (as a standalone device) using the built-in viewer capable of playing 360° content. This setup was selected based on the results of previous research supporting the use of combined audiovisual inputs via a head-mounted display to induce the greatest relaxation effect (17, 30). The device had a resolution of 3,840 × 2,160 pixels, with a refresh rate of 75 Hz and a weight of 276 g. The audio content was played using a wired connection to a set of binaural over-ear noise-canceling headphones (Sony WH-1000XM3; Sony Group Corp., Minato City, Tokyo, Japan). The content was played back at the preferred participant volume, with the participants being able to further adjust the volume themselves.

### 2.4. Statistical analysis

The patient data management system stored the data at a frequency of one sample per 2 min. For continuous measurements, the data stored was the median of the recorded values within 2 min, which helped reduce artifacts. Data extraction from the existing databases also resulted in dropped samples. Data dropped at single timepoints were interpolated using a third-order polynomial (31). Variables that were missing more than one subsequent timestamp or missing data at the beginning or end of the VR sessions were discarded. Consequently, all participants whose blood pressure was measured using a non-continuous cuff-based monitor were excluded from the analysis, as there were not enough samples recorded to provide a reliable assessment. Only the mean arterial blood pressures were analyzed for this study.

For data analysis, descriptive statistics were applied for the participants’ demographics and severity of disease scores. Specifically, the Acute Physiology and Chronic Health Enquiry (APACHE II) represents a validated severity of disease classification system, while the Simplified Acute Physiology Score (SAPS II) provides the risk of death without providing a diagnosis (32, 33). A simple linear regression and Pearson’s correlation were applied to the median of the 2-min intervals to investigate what kind of change, if any, occurred in the heart rate and blood pressure during each of the VR sessions. The data were weighted per time point to account for dropouts after the first 20 min of the intervention. Next, based on the information obtained from the model, the line parameters (i.e., slope and intercept) of the population were further examined to investigate measurement errors and trends within the data. This goal was achieved by examining the differences between the values of each parameter (i.e., heart rate and blood pressure at the start and end of each VR session) compared with their mean using Bland–Altman plots. Within Bland–Altman plots, there are seven categories that can be used for the interpretation. In total, three categories represent a positive effect (i.e., an increase in the physiological value); three categories, a negative effect (i.e., a decrease in the physiological value); and one category, a fixed bias zone reflecting the random fluctuations around the mean. Values within this fixed bias zone represent those for which an effect with certainty (i.e., positive or negative) cannot be clearly stated. Within each positive and negative zone, there are: (1) the main effect zone located between the bias and the limits of agreement, (2) the limits of agreement and their 95% confidence interval (CI), and (3) the outlier zone found beyond the limits of agreement. In this study, Bland–Altman plots were also used to detect the presence of any proportional biases within the bounds of the CI that could influence the data.

The similarity was calculated between the mean of the population at each timepoint to investigate the relationship between the heart rate and blood pressure during each session. Additionally, Pearson’s correlation coefficients were computed to assess the linear relationship between the blood pressure and heart rate during each VR session.

The data collected via the questionnaire were examined and presented as medians and interquartile ranges (IQR). A non-parametric Friedman test was used to determine whether there were any significant changes in the pain score before and after the VR session. Significant results were followed up using the pairwise Wilcoxon signed-rank test to identify which pain scores differed after correcting for multiple comparison factors using Bonferroni adjustment. All preprocessing and statistical analyses were completed using Python 3.10.9 (Python Software Foundation, Wilmington, Delaware, USA) and R for Statistics 4.2.3 (The R Foundation, Vienna, Vienna, Austria).

## 3. Results

### 3.1. Demographics

**Figure 2:**
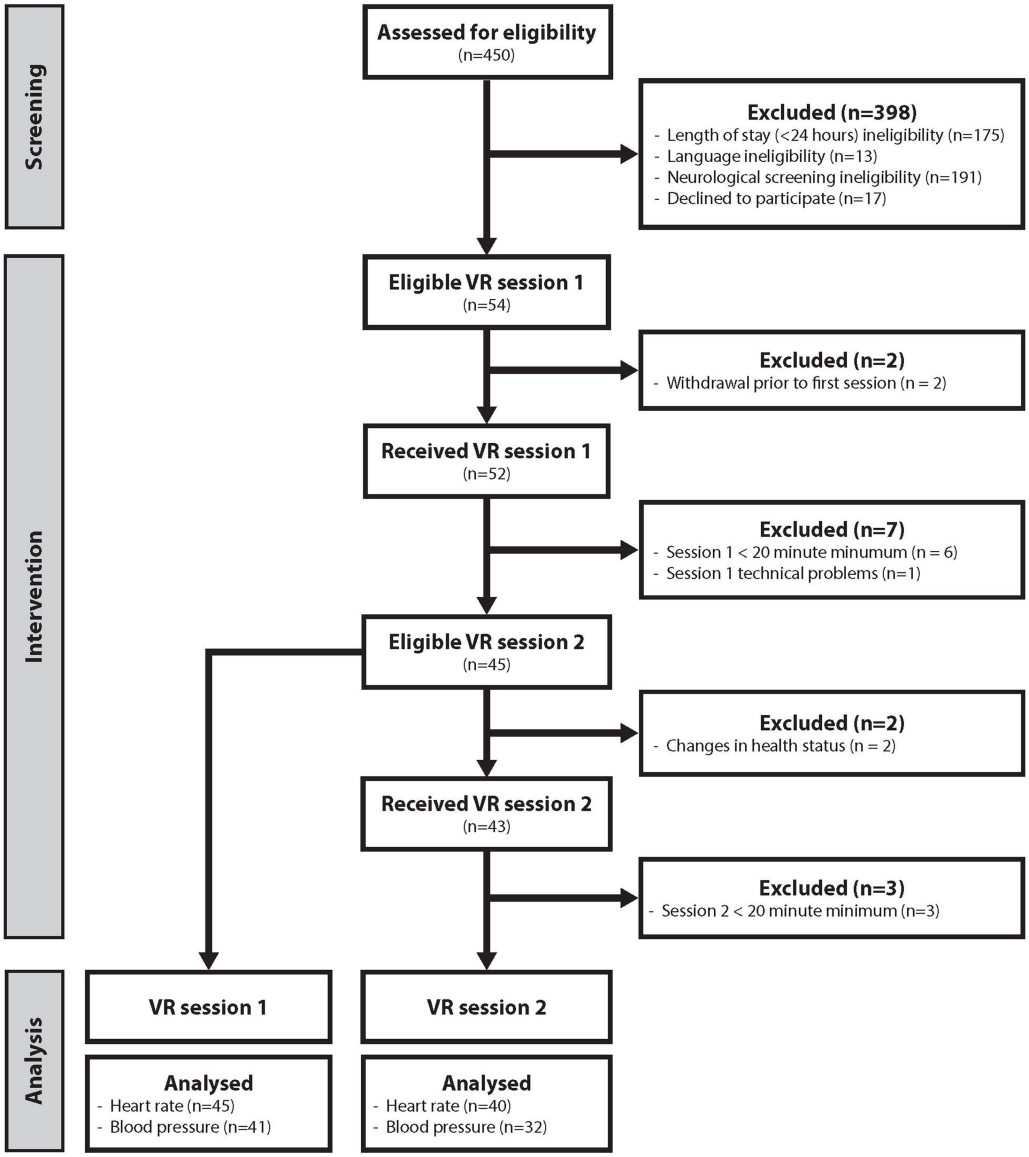
Enrollment schematic. Flowchart depicting the workflow from patient screening and enrollment and intervention to the final analyses. Neurological screening ineligibility means that a participant met at minimum one of the neurology-based exclusion criteria (i.e., epilepsy, skullcap, external ventricular drainage, intracranial probe). Lesser blood pressure data were analyzed, as the cuff-based data were excluded from the analyses. VR, virtual reality.

In total, 54 (21 women, 33 men, age = 57.24 ± 15.85 years) patients hospitalized in the IMCU were enrolled in the study. Among them, 45 (20 women, 25 men, age = 57.73 ± 15.92 years) completed the first VR session and were included in the final analysis. Of the nine patients with datasets that were discarded, two withdrew prior to the first session; six had an incomplete first session (duration of <20 min); and one had an incomplete dataset owing to a technical problem (Figure 2). Of the two patients who withdrew prior to the first session, one did so as they were having trouble coming to terms with their diagnosis, whereas the second was worried about the HMD being uncomfortable due to the placement of their wound on their head. Of the six who had incomplete first sessions, these participants all chose to end the session prematurely themselves; two had simply wanted to test the technology, three were overwhelmed and fatigued by the study, and one did not realize the study team would inform them when the minimum 20 min was completed. The 6 participants who did not complete the first session had the following durations: 4, 4, 5, 7, 12, and 13 min.

Of the patients who completed the first session, two did not start the second session owing to changes in their health status, and three started the second session, but which was incomplete (duration of <20 min) (Figure 2). The reasons for not starting the second session included pain from a wound on the back of the head making wearing of the device undesirable and a general deterioration in health making study participation too overwhelming. The three incomplete sessions were ended prematurely and independently by the participant; one was due to discomfort, while two were due to the participants believing they had seen enough. The 3 participants that did not complete the second session had the following durations: 8, 17, and 17 min. In total, 40 (16 women, 24 men, age = 57.55 ± 15.97 years) participants completed the second VR session and were included in the final analysis.

At the time of each VR session, the participants had a Glasgow Coma Score of 15. During the first 24 h of their stay in the ICU, the participants had an average APACHE II score of 10.53 (*n* = 40; 95% CI = 9.17–11.87) and an average SAPS II score of 21.67 (*n* = 42; 95% CI = 18.43–24.91). The admission classification and diagnosis are shown in Table 1. All participants were extubated, and none received sedation medication. During the first session, 15.91% of the participants received medication influencing the heart rate; 29.55%, medication influencing the blood pressure; and 70.45%, pain medication. During the second session, no participants received medication influencing the heart rate; 27.50% received medication influencing the blood pressure; and 75.00% received pain medication.

**Table 1:** Admission classification and diagnosis.

Admission classification		Admission diagnosis	
Emergency	*n* = 14	Neurological - Intracerebral Bleeding - Tumor - Stroke - Other	*n* = 28
Planned Admission	*n* = 7		11
			6
			2
Central Recovery Room	*n* = 1		9
External Hospital	*n* = 1	Gastrointestinal - Tumor - Organ Transplant - Other	*n* = 10
			5
			1
			4
Planned Surgery	*n* = 18	Sepsis	*n* = 2
Unplanned Surgery	*n* = 2	Trauma - Multiple Trauma	*n* = 3
			3
Invasive Other	*n* = 2	Other	*n* = 2

### 3.2. Time effect on physiological parameters

**Figure 3:**
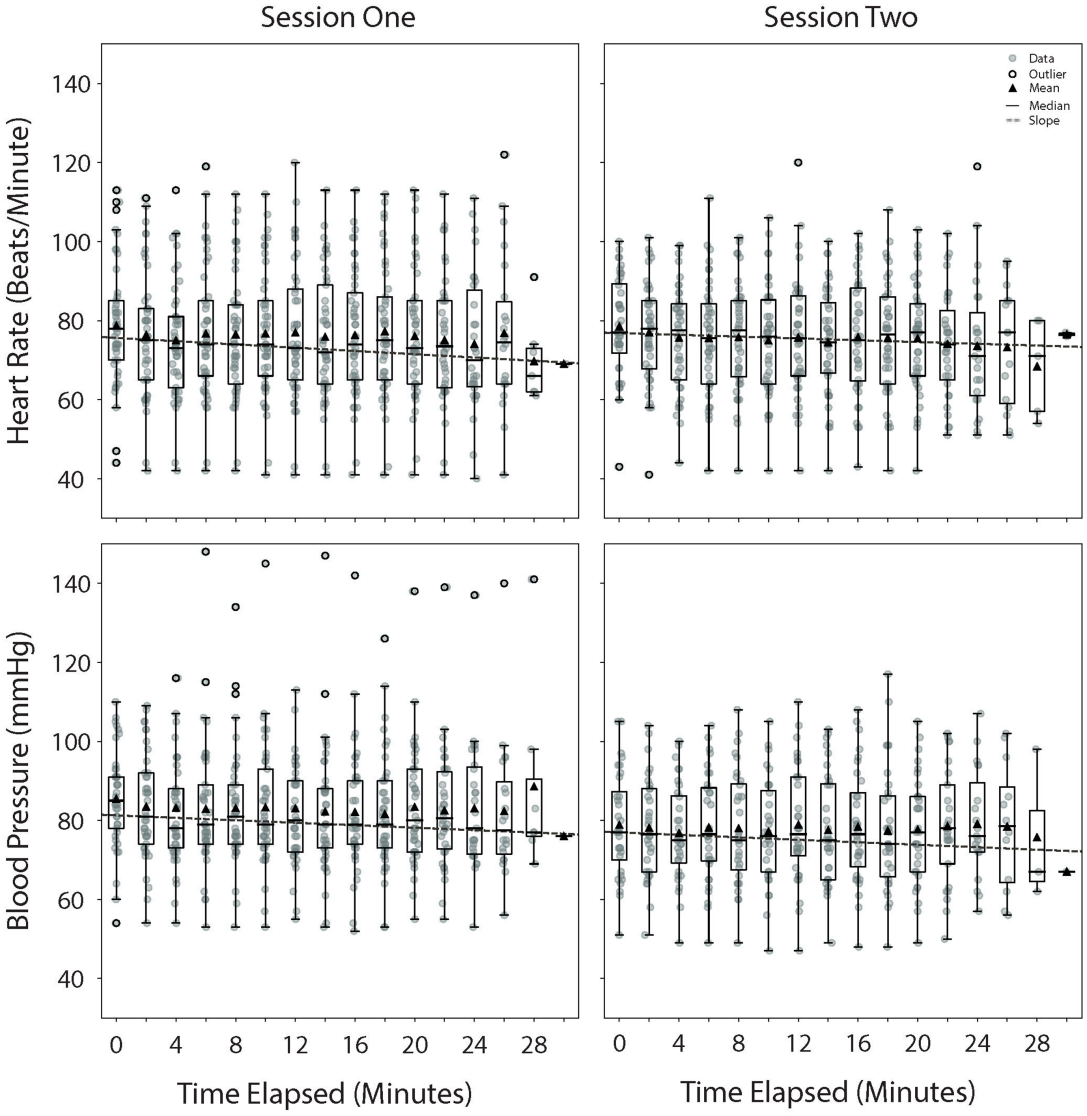
Physiological change throughout the VR sessions. Heart rate (top) and blood pressure (bottom) throughout the VR sessions. Time 0 represents the start of the session. The average duration of the sessions was 24 ± 2.62 min. The mean of the data points per time period is represented by a triangle symbol. VR, virtual reality.

First session: The mean (± standard deviation) heart rate 10 min prior to and at the session start was 78.32 ± 15.32 and 78.71 ± 15.11 beats per minute, respectively. At 10 min and 20 min into the stimulation, the mean heart rate was 76.74 ± 16.08 and 76.04 ± 16.18 beats per minute, respectively (Figure 3). In the investigation of the relationship between the heart rate and stimulation duration, we found a strong negative correlation [*r*(43) = −0.78, *p* < 0.001] between the parameters. This suggests that as the duration of the stimulation increased, there was a tendency for the heart rate to decrease, and this relationship was statistically significant. A linear relationship was found (slope = −0.41), with a good coefficient of determination (R^2^ = 0.61) (34). In terms of the heart rate, a total of 31 participants had a slope more negative than 0; 14 had a slope more positive than 0; and none had a slope equal to 0 (Supplementary Figure S1).

The mean blood pressure 10 min prior to and at the session start was 84.88 ± 11.76 and 85.46 ± 12.27 mmHg, respectively. At 10 min and 20 min into the stimulation, the mean blood pressure was 83.31 ± 15.92 and 83.12 ± 14.92 mmHg, respectively (Figure 3). There was a strong negative correlation [*r*(39) = −0.78, *p* < 0.001] between the blood pressure and stimulation duration. This suggests that as the duration of the stimulation increased, there was a tendency for the blood pressure to decrease, and this relationship was statistically significant. A linear relationship was determined between these variables (slope = −0.31), with a moderate coefficient of determination (R^2^ = 0.51). In terms of the blood pressure, a total of 27 participants had a slope more negative than 0; 14 had a slope more positive than 0; and none had a slope equal to 0 (Supplementary Figure S1).

Second session: The mean heart rate 10 min prior to and at the session start was 78.25 ± 14.09 and 78.47 ± 12.54 beats per minute, respectively. At 10 min and 20 min into the stimulation, the mean heart rate was 74.93 ± 13.92 and 75.50 ± 13.58 beats per minute, respectively (Figure 3). In the investigation of the relationship between the heart rate and stimulation duration, we found a strong negative correlation [*r*(38) = −0.81, *p* < 0.001] between the parameters. This suggests that as the duration of the stimulation increased, there was a tendency for the heart rate to decrease, and this relationship was statistically significant. A linear relationship was found (slope = −0.23), with a weak coefficient of determination (R^2^ = 0.27). During the session, 25 participants had a slope more negative than 0; 15 had a slope more positive than 0; and none had a slope equal to 0 (Supplementary Figure S1).

The mean blood pressure 10 min prior to and at the session start was 81.84 ± 16.45 and 78.53 ± 12.79 mmHg, respectively. At 10 min and 20 min into the stimulation, the mean blood pressure was 77.16 ± 13.02 and 77.66 ± 13.40 mmHg, respectively (Figure 3). There was a strong negative correlation [*r*(30) = −0.78, *p* < 0.001] between the blood pressure and stimulation duration. This suggests that as the duration of the stimulation increased, there was a tendency for the blood pressure to decrease, and this relationship was statistically significant. A linear relationship was determined between these variables (slope = −0.31), with a weak coefficient of determination (R^2^ = 0.21). During the session, 16 participants had a slope more negative than 0; 16 had a slope more positive than 0; and none had a slope equal to 0 (Supplementary Figure S1).

The effect of the VR stimulation, measured as the change from the start to the end of the session, was found to result in a mean effect of −1.24 ± 1.50 beats per minute (*n* = 45) for the heart rate and −1.98 ± 1.45 mmHg (*n* = 41) for the blood pressure during the first session. Conversely, there was a mean effect of −2.25 ± 1.42 beats per minute (*n* = 40) for the heart rate and −0.47 ± 1.49 mmHg (*n* = 32) for the mean arterial pressure during the second session (Supplementary Figure S2). The full details per zone are shown in Table 2. Based on the Bland–Altman plots, no proportional bias was observed for any variable in either VR session (Supplementary Figure S2).

**Table 2:** Effect of VR from start to end of each VR session.

	Heart rate				Mean blood pressure			
	Session 1		Session 2		Session 1		Session 2	
	No. of Samples	Mean of samples (beats/min)	No. of samples	Mean of samples (beats/min)	No. of samples	Mean of samples (mmHg)	No. of samples	Mean of samples (mmHg)
Positive (Increasing) effect	6	6.00 ± 3.26	9	3.44 ± 2.79	7	4.71 ± 2.91	9	5.22 ± 2.39
Negative (Decreasing) effect	17	−8.47 ± 2.28	14	−7.79 ± 1.66	19	−8.11 ± 2.67	5	−5.2 ± 1.60
Bias zone	20	−1.10 ± 1.55	15	−2.52 ± 1.62	12	−2.33 ± 1.37	13	−0.08 ± 1.38
Upper limit of agreement	0	N/A	0	N/A	2	15.00 ± 3.00	2	14.00 ± 3.00
Lower limit of agreement	0	N/A	1	−17.00 ± 0.00	0	N/A	2	−20.0 ± 2.00
Positive outliers	2	37.00 ± 8.00	1	43.00 ± 0.00	1	38.00 ± 0.00	0	N/A
Negative outliers	0	N/A	0	N/A	0	N/A	1	−23.00 ± 0.00

The number and mean (± standard deviation) of the samples were categorized on the basis of their change from the start to the end of each VR session. N/A indicates categories in which no samples were found, and no mean was calculated accordingly. VR, virtual reality.

#### 3.2.1. Relationship between the heart rate and blood pressure

The value of the relationship between the heart rate and blood pressure during the first and second sessions was both 0.99, respectively. The Pearson’s correlation coefficient between the two variables was 1.00 during the first (*p* < 0.001) and second sessions (*p* < 0.001).

### 3.3. Questionnaire data

**Figure 4:**
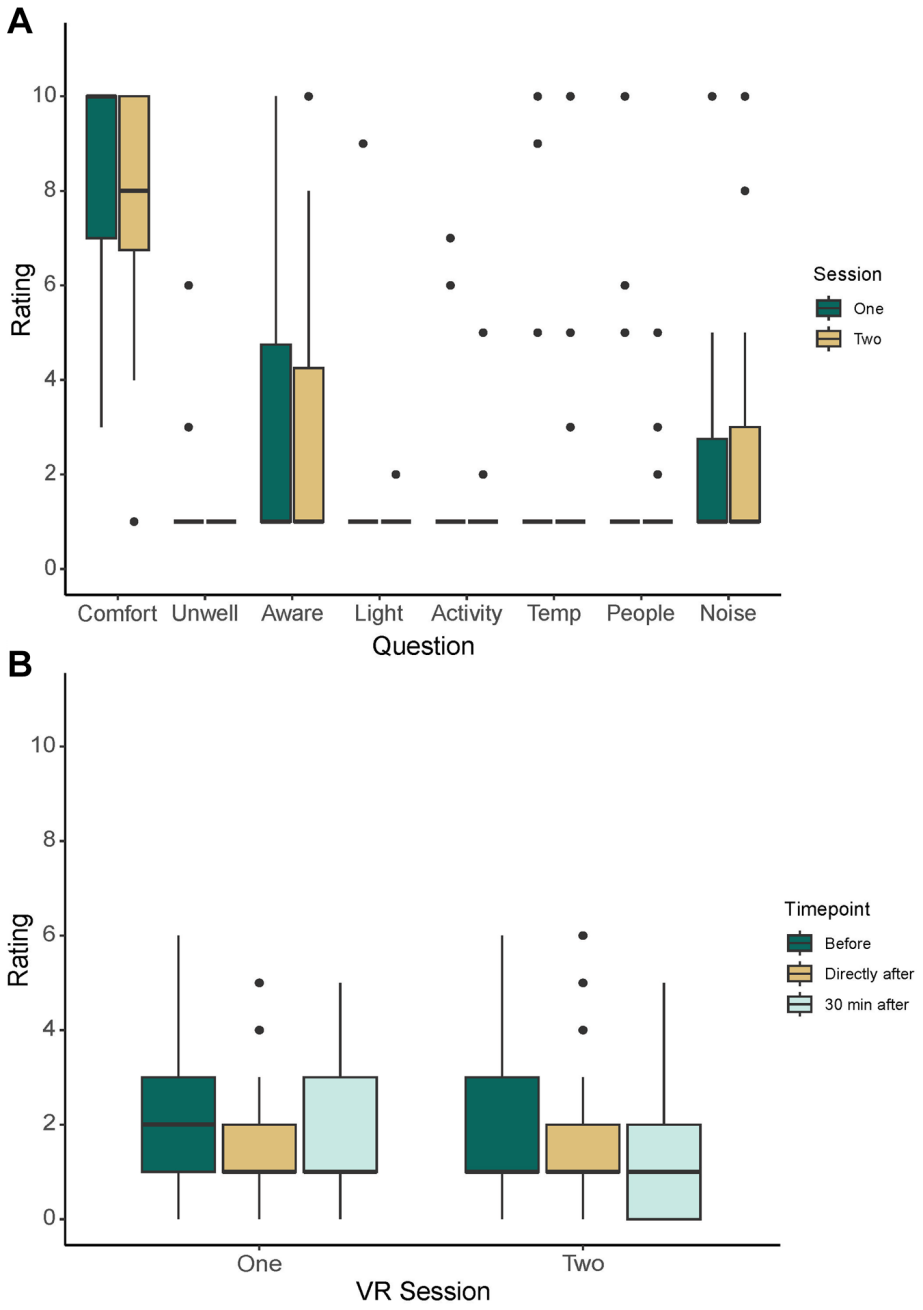
General Awareness and Pain Scores. General comfort and awareness scores **(A)** as reported via questionnaire following VR sessions one and two. Pain scores **(B)** as reported using the Numeric Rating Scale immediately before, directly after, and 30 min after each VR session. VR, virtual reality.

The findings from the questionnaire revealed that the median comfort score was 8 (IQR = 7, 10; mean = 8.06 ± 2.31) and that the participants did not report being unwell (e.g., nausea, dizziness, malaise) during the sessions [median (IQR) = 1 (1, 1); mean = 1.11 ± 0.62] (Figure 4A). The participants also reported not being substantially aware of their real-world surroundings during the VR sessions with a median score of 1 (IQR = 1, 5; mean = 2.99 ± 3.22) (Figure 4A). Specifically, the participants were not at all aware of the light [median (IQR) = 1 (1, 1); mean = 1.10 ± 0.87], activity [median (IQR) = 1 (1, 1); mean = 1.20 ± 0.94], temperature [median (IQR) = 1 (1, 1); mean = 2.07 ± 2.71], and people [median (IQR) = 1 (1, 1); mean = 1.49 ± 1.49] in the room during the VR stimulation (Figure 4A). They were only slightly more aware of the noise [median (IQR) = 1 (1, 3); mean = 2.25 ± 2.20] (Figure 4A). During the first and second VR sessions, 60.00% (*n* = 27/45) and 62.50% (*n* = 25/40) of the participants reported falling asleep at least once, respectively. No adverse events or serious adverse events were reported by the patients or care staff within 2 h following the VR sessions.

There were no significant differences in the pain scores before [median (IQR) = 1 (1, 3); mean = 1.91 ± 1.43] and immediately [median (IQR) = 1 (1, 2); mean = 1.68 ± 1.22] and 30 min after the first session [median (IQR) = 1 (1, 3); mean = 1.75 ± 1.38] [χ^2^_Friedman_(2) = 1.02, *p* = 0.601] (Figure 4B). Conversely, there were significant differences in the pain scores before [median (IQR) = 1 (1, 1); mean = 2.00 ± 1.66] and immediately [median (IQR) = 1 (1, 1); mean = 1.68 ± 1.42] and 30 min after the second session [median (IQR) = 1 (1, 1); mean = 1.60 ± 1.66] [χ^2^_Friedman_(2) = 6.58, *p* = 0.0372] (Figure 4B). The post-hoc analysis revealed significant differences in the scores before and immediately after the sessions (*p* = 0.025) and before and 30 min after the sessions (*p* = 0.039).

## 4. Discussion

In this study, the aim was to investigate how a random sample of critically ill individuals objectively and subjectively react to repeated sessions of immersive VR. In line with our first hypothesis, this study showed a small but significant decrease in the heart rate and blood pressure during the VR sessions. Moreover, our results also supported our second hypothesis, finding that the participants rated the wearing of the head-mounted display and headphones as comfortable while being generally less aware of their real-world surroundings during the VR sessions. Specifically, the participants reported being the least aware of light, activity, and people in the hospital room, highlighting the potential use of immersive VR for addressing sensory overload and sensory deprivation in the ICU.

While sensory overload typically occurs more frequently in the ICU setting than sensory deprivation, the application of these findings is relevant to both extremes. This work has shown that the real-world surroundings can be blocked using immersive VR, therefore, it can be hypothesized that this technology will be equally successful in improving patient experiences whether it is for sensory overload or deprivation. Moreover, the natural fluctuation of activity and patients in the study setting meant that not all immersive VR sessions occurred during periods of high sensory inputs.

### 4.1. Physiological effects

The physiological effects observed in this study align with existing literature on the topic. Previous work examining the relaxation effect of immersive VR has found a decrease in physiological parameters, namely heart rate, respiration rate, and blood pressure (17, 18). The heart rate and blood pressure data collected herein align with previous work, with the two variables showing a significant agreement and correlation with each other, suggesting the presence of a similar negative trend between them. Accordingly, the clinical significance of such decreases in parameters and what type of diagnosis is most associated with these decreases warrant further research. Moreover, the results of the Bland–Altman analysis in this study suggest that not all patients may benefit equally, with some even having an increase in their physiological heart rate and blood pressure. However, this does not automatically indicate a negative reaction to the immersive VR. Indeed, an increase in these parameters could have potentially arisen as some participants actively explored the virtual environment, moving their entire bodies to do so. Another explanation could be linked to the fact that sometimes nurses had to adjust medical equipment during the running of the experiment when patients were in a more upright seated position; certain changes are known to increase the blood pressure and should be controlled for in future studies. However, despite this increase, providing immersive VR to patients as a tool for preventing or treating sensory overload should not be dismissed considering the positive responses obtained via the questionnaire. The Bland–Altman analysis data also suggest that the changes could be clinically relevant for patients who experience a decrease in their blood pressure or heart rate.

The present results provide evidence that the relaxation effect seen in healthy individuals and critically ill patients with prior exposure to immersive VR can be replicated in a random sample, covering a range of diagnoses. This suggests that immersive VR has the ability to be widely applicable in the ICU and provides evidence that this technology can be successfully implemented to address sensory overload and sensory deprivation. For this purpose, further work creating content specific to each extreme (i.e., overload versus deprivation) should be carried out. Doing so could also allow for more targeted sensory inputs to be provided, specifically with the goal of re-orienting patients, which can beneficially impact them (4, 35–37).

### 4.2. Questionnaire data

The physiological data obtained were further supported by the questionnaire data, showing a strong potential for immersive VR to be used to address sensory overload and sensory deprivation. Our participants not only found the setup comfortable but were also less aware of their surroundings, a key problem in sensory overload (3–5). The participants were most aware of noise during the VR sessions; however, this awareness was still minimal. Alternatives to addressing sensory overload and sensory deprivation presented in the literature focus on patient-centered lighting via personal ICU lighting systems to help combat delirium in the ICU by reproducing more naturalistic light cycles (38, 39). However, the clear disadvantage of such systems is that they do not allow for a visual or auditory respite. As such, it is possible that patients remain aware of all comings and goings of healthcare professionals, both to their bed and other beds, as well as all activities at neighboring beds. This issue can only be addressed by altering the visual field of patients while blocking unwanted auditory inputs. As found in previous work, this goal is best achieved by providing concurrent visual and auditory stimuli (17).

Another aspect to consider based on the present results is the usefulness of using immersive VR in the treatment of pain. This effect has long been explored in the literature, with many studies highlighting the usefulness of VR as a pain reduction tool (40, 41). In contrast to previous studies, our study found a significant reduction in the pain scores from pre-to post-VR stimulation in only one of the two VR sessions. However, despite its statistical significance, the minimum change to indicate a clinically relevant reduction, typically referred to as the minimally clinically important difference (MCID), is likely not reached. Studies on different types of pain using the Numeric Rating Scale typically put the MCID between 1–2.17 (42–45). However, pain and pain reduction remains subjective and depends on the sensitivity of the patient (46). As such, it is difficult to make generalizations about the efficacy of immersive VR as a pain reduction tool for all patients in this setting. Moreover, potential efficacy may have been masked by the high percentage of critically ill patients who received pain medication, indicating that their pain scores were already being managed.

An alternative explanation for the decrease in reported pain levels could be related to the fact that critically ill patients often report not sleeping well enough, which is known to be associated with an increased perception of pain (47, 48). With over half of the participants of this study reporting that they fell asleep at least once during the VR sessions, there could be a relationship between the use of VR, sleep, and the subjective rating of pain. Therefore, the use of VR as a tool for promoting sleep by controlling sensory overload and consequently reducing pain could be further explored in future work (49).

### 4.3. Limitations and future outlooks

A limitation of the study is related to the fact that some patients were restricted in their ability to turn on their beds. This means that these patients were not able to fully explore the virtual environment. This restriction must be considered when creating VR contents to ensure that enough interest-generating activity can be viewed without individuals having to turn their head. Alternatively, the feasibility of having patients with limited mobility explore their surroundings via a controller could also be investigated.

Another aspect that could be further refined in future work is the duration of the videos and any influence it could have on the physiological effect seen herein. In this study, nine participants had unsuccessful VR sessions, meaning that they did not complete a 20-min VR session. Therefore, the effect, beneficial or otherwise, was not examined. Accordingly, it is possible that certain patients may benefit from repeated shorter adaptation sessions, building up to longer sessions, or, alternatively, the freedom to determine the duration of the stimulation themselves.

Despite the limitations presented here, this study provides evidence that not only would immersive VR technology be well accepted in critically ill patients, but that it can potentially be integrated into routine care as a tool to combat sensory overload and sensory deprivation in this setting. In doing so, this work opens new avenues for future work and VR companies. For example, this technology has the possibility to improve long term patient quality of life after hospital discharge by improving their ICU stay. In this way, the potential benefit to patients could be quite important and warrants further research. Another application that could positively benefit patients and healthcare staff, and is worth future research, is the use of immersive VR as a prevention strategy for delirium in ICU patients (50). With its many potential uses, a growing number of VR technology companies are recognizing the various use cases for immersive VR within the clinical setting and providing solutions targeting not only pain and anxiety relief, but cognitive training as well (e.g., Super Sublime, Healthy Mind, SenopiVR). As such, the current work acts as a valuable stepping stone to help these companies bring immersive VR technology to the clinics and, more importantly, to the patients.

## 5. Conclusion

This study provides evidence that immersive VR can successfully be repeatedly provided to a wide variety of critically ill patients in the ICU and can decrease their awareness of their real-world surroundings during VR sessions. Moreover, this study shows a small but significant decrease in the heart rate and blood pressure during both stimulation periods, suggesting the possibility of an induction of a relaxation effect in the population. Therefore, immersive VR shows a potential to be a valuable tool in addressing sensory overload and sensory deprivation in the ICU.

## Data availability statement

The datasets for this article are not publicly available due to concerns regarding participant/patient anonymity. Requests to access the datasets should be directed to the corresponding author.

## Ethics statement

The studies involving humans were approved by Ethics Committee of the Canton of Bern, Switzerland (KEK-2020-00039b). The studies were conducted in accordance with the local legislation and institutional requirements. The participants provided their written informed consent to participate in this study.

## Author contributions

AN: Conceptualization, Data curation, Formal analysis, Investigation, Methodology, Project administration, Visualization, Writing – original draft, Writing – review & editing. SG: Conceptualization, Methodology, Visualization, Writing – original draft, Writing – review & editing. MS: Formal analysis, Methodology, Visualization, Writing – original draft, Writing – review & editing. RM: Conceptualization, Supervision, Writing – review & editing. MH: Project administration, Resources, Supervision, Writing – review & editing. SJ: Conceptualization, Investigation, Project administration, Resources, Supervision, Writing – review & editing. M-MJ: Conceptualization, Investigation, Methodology, Project administration, Resources, Supervision, Writing – review & editing. TN: Conceptualization, Project administration, Resources, Supervision, Writing – review & editing.
