# Supplementary material for: Effects of immersive virtual reality on sensory overload in a random sample of critically ill patients
Source: Front Med (Lausanne). 2023 Oct 4;10:1268659. doi: 10.3389/fmed.2023.1268659 (PMC10582722; doi:10.3389/fmed.2023.1268659)
Supplement: Supplementary file 1 [file Data_Sheet_1.PDF]

Supplementary Material

Effects of Immersive Virtual Reality on Sensory Overload in a Random Sample of Critically Ill Patients

1 Supplementary Tables

1.1 General Awareness Questionnaire

Supplementary Table S1. General Awareness Questions. Participants were asked to answer the following questions (Q) immediately after each stimulation session.

		Not at all				Somewhat					Extremely
**Q1.**	I found the VR system to be comfortable	☐	☐	☐	☐	☐	☐	☐	☐	☐	☐
**Q2.**	During my experience I felt unwell (e.g., nausea, dizziness, etc. ...)	☐	☐	☐	☐	☐	☐	☐	☐	☐	☐
**Q3.**	I was aware of my real surroundings	☐	☐	☐	☐	☐	☐	☐	☐	☐	☐
**Q4.**	I was aware of the following things:										
**4a)**	Light *(warning signals, room lighting, ...)*	☐	☐	☐	☐	☐	☐	☐	☐	☐	☐
**4b)**	Noise *(sounds of equipment, alarms, opening of packages, ...)*	☐	☐	☐	☐	☐	☐	☐	☐	☐	☐
**4c)**	Temperature *(warm, cold, draught, ...)*	☐	☐	☐	☐	☐	☐	☐	☐	☐	☐
**4d)**	Restlessness/stress *(activity or hecticness on the ward, ...)*	☐	☐	☐	☐	☐	☐	☐	☐	☐	☐
**4e)**	Staff *(coming and going, conversation...)*	☐	☐	☐	☐	☐	☐	☐	☐	☐	☐

2 Supplementary Figures

2.1 Slope Distribution

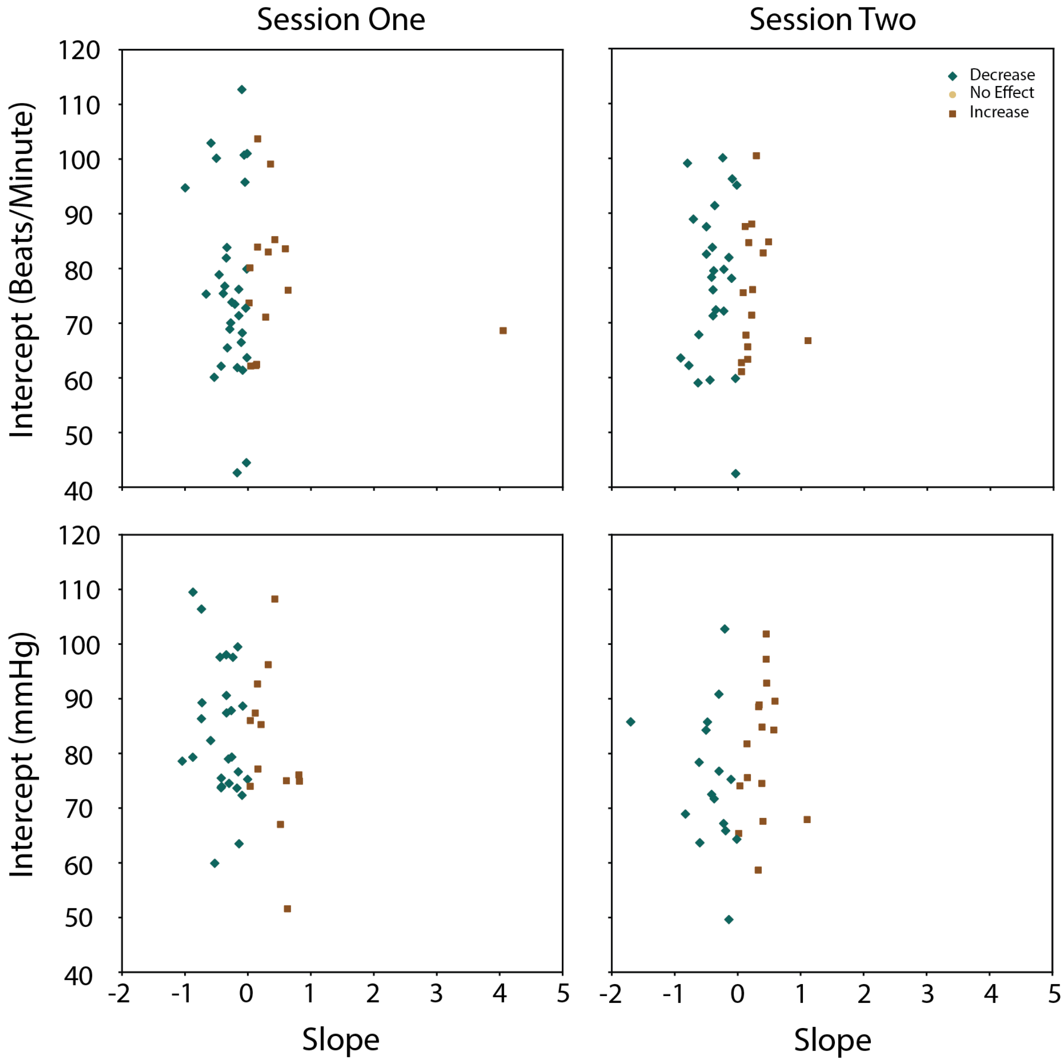

Supplementary Figure S1. Linear Regression Slope Distribution. Distribution of the change in heart rate and blood pressure during each VR session. Turquoise diamonds indicate slopes more negative than zero, indicating a decrease in the physiological value; yellow circles indicate slopes equal to zero, indicating no change in the physiological value; brown squares indicate slopes more positive than zero, indicating an increase in the physiological value.

2.2 Bland Altman

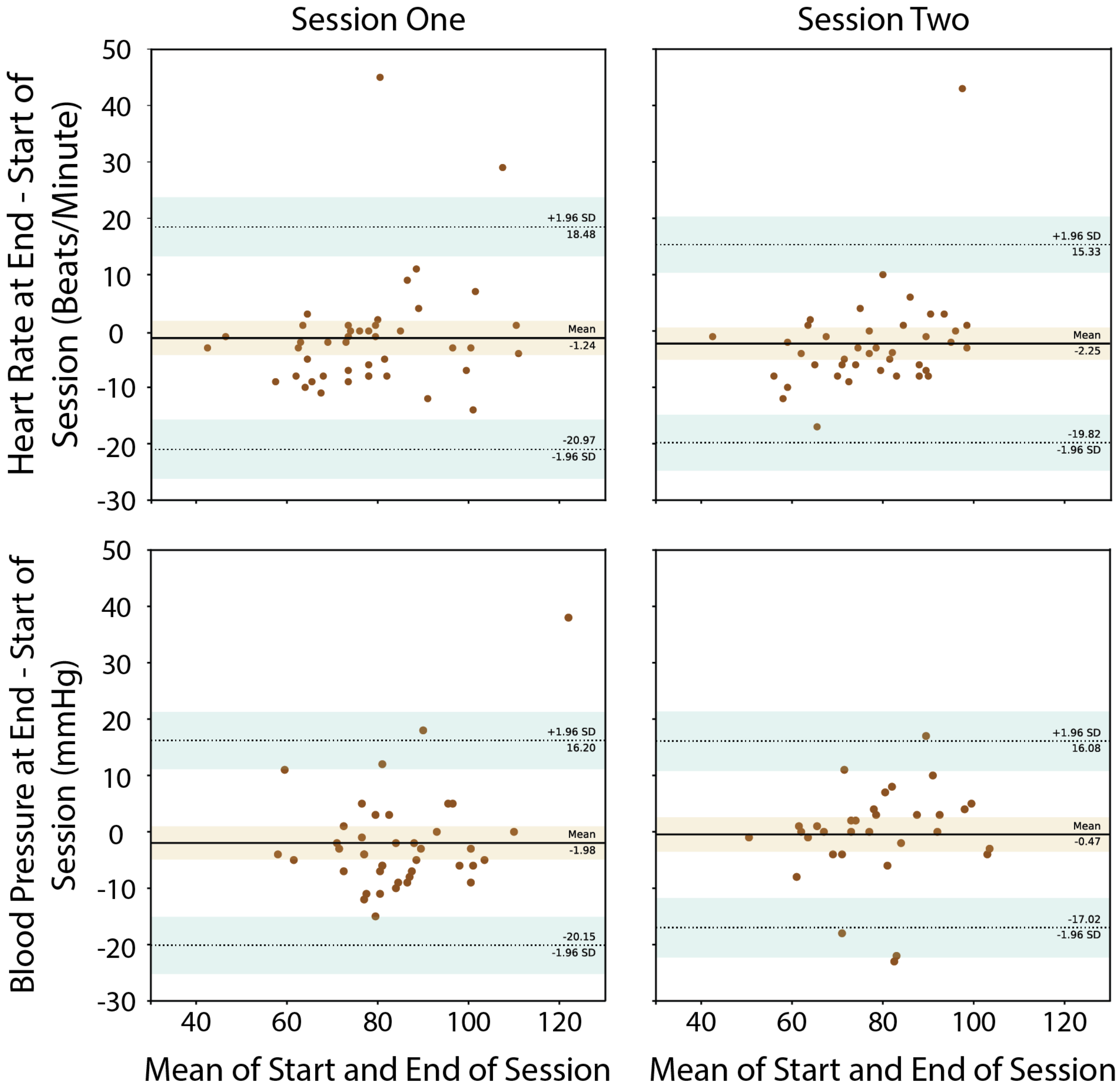

Supplementary Figure S2. Bland Altman Plots. Results from the Bland Altman analysis showing the various effect zones per VR session for heart rate (top) and blood pressure (bottom). The beige band represents the bias zone, while the blue bands represent the upper and lower limits of agreement.
